# A quantitative model of cellular decision making in direct neuronal reprogramming

**DOI:** 10.1038/s41598-021-81089-8

**Published:** 2021-01-15

**Authors:** Adriaan Merlevede, Emilie M. Legault, Viktor Drugge, Roger A. Barker, Janelle Drouin-Ouellet, Victor Olariu

**Affiliations:** 1 Computational Biology and Biological Physics, Department of Astronomy and Theoretical Physics, Lund University, 223 62 Lund, Sweden; 2 Cambridge Centre for Brain Repair, University of Cambridge, Forvie Site, Robinson Way, Cambridge, CB2 2PY UK; 3 Faculté de Pharmacie, Université de Montréal, Montreal, QC H3T 1J4 Canada

**Keywords:** Gene regulatory networks, Computational biology and bioinformatics, Nonlinear dynamics, Numerical simulations, Stochastic modelling, Systems biology, Time series, Transdifferentiation, Transdifferentiation

## Abstract

The direct reprogramming of adult skin fibroblasts to neurons is thought to be controlled by a small set of interacting gene regulators. Here, we investigate how the interaction dynamics between these regulating factors coordinate cellular decision making in direct neuronal reprogramming. We put forward a quantitative model of the governing gene regulatory system, supported by measurements of mRNA expression. We found that nPTB needs to feed back into the direct neural conversion network most likely via PTB in order to accurately capture quantitative gene interaction dynamics and correctly predict the outcome of various overexpression and knockdown experiments. This was experimentally validated by nPTB knockdown leading to successful neural conversion. We also proposed a novel analytical technique to dissect system behaviour and reveal the influence of individual factors on resulting gene expression. Overall, we demonstrate that computational analysis is a powerful tool for understanding the mechanisms of direct (neuronal) reprogramming, paving the way for future models that can help improve cell conversion strategies.

## Introduction

Cell differentiation, the process that establishes cellular identity, traditionally follows a well-established hierarchy from totipotent to nilpotent cell types. However, the past decade has seen great advances in reprogramming procedures, allowing researchers to convert terminally differentiated cells to other cell types in vitro. This technology has promising applications for disease modelling, where an individual’s skin fibroblasts can be converted to neurons and be used to determine their own unique neuronal pathology in vitro, as well as regenerative medicine, where replacement tissues can be sourced from a patient’s own body. Direct reprogramming, in particular, allows conversion between terminally differentiated cell types without passing through an intermediate pluripotent state. This gives the opportunity to obtain mature neurons quickly (within a month) and allowing for relatively easy handling of dozens of lines by one experimenter. Moreover, they are not clonal, thus avoiding the risk of clonal bias. Importantly, multiple reports have recently demonstrated that directly reprogrammed neurons retain many important aspects of the age signatures of the donor, including age-related changes in the epigenetic clock, the transcriptome and microRNAs, the reactive oxygen species (ROS) levels, DNA damage and telomere lengths, as well as the metabolic profile and mitochondrial defects^1–4^. As these cellular changes are suspected to play crucial roles in the development of age-associated disorders, this makes direct neuronal reprogramming an ideal approach to model neurodegenerative diseases for which age is the most important risk factor. However, because the source cells do not pass through a proliferating intermediate stage, a high conversion efficiency is required for large scale clinical application.

The working principle of contemporary reprogramming methods is to introduce a select combination of transcription factors and other biomolecules that manipulate the expression of key genes, initiating a cascade of regulatory mechanisms that control all known aspects of cell identity. Finding a combination of appropriate factors that induce the desired conversion with sufficient efficiency is the central difficulty for current reprogramming research, and is typically done by trial and error, using a set of candidate genes picked from databases and published resources based on the characteristic expression profiles of the source and target cell types. Some effort has gone into streamlining this process of selecting candidate genes^5^, but the efficiency of these tools is bounded by our knowledge of the underlying gene interactions.

One of the most notable cases is conversion of somatic dermal cells into neurons, which shows great promise in the study and treatment of neurodegenerative disorders^6^. Dermal fibroblasts are highly suitable source cells because they are abundant and easy to harvest. Many different cocktails of transcription factors have been successfully applied. Originally, Brn2, Ascl1 and Myt1l were shown to be sufficient in adult mouse fibroblasts^7^. In humans, homologous factors could induce neural conversion in fibroblasts at a very low efficiency^8^, which could be improved by the addition of other factors such as Ngn2, Sox2, NeuroD1/2, miR-9/9*, and/or miR-124^6,9,10^. The efficiency of some of these methods has been increased by concomitant knockdown (KD) of the REST complex^6,10,11^. Another approach involves knockdown of PTB^12,13^. Other factors have been used to convert to specific neural subtypes^9^.

The experimental successes in the field of neural reprogramming indicate that, although the specialized identity of the cell constitutes many different aspects of cellular form and function, it is fundamentally controlled by only a few key regulators. Much interest has gone to identifying these key regulators and their roles, but the attempts to integrate this knowledge into a holistic understanding of the gene interaction network are still in the early stages. A quantitative model that captures the essential properties of the neural conversion mechanism represents an invaluable tool for suggesting new experiments and more efficient reprogramming methods. Researchers have engaged in such modelling in closely related fields such as the study of pluripotent cell commitment and reprogramming somatic cells to pluripotency. The resulting models successfully compress many experimental results into a single framework that can be interpreted by researchers, provides further experimental predictions, and can be used to suggest new experiments^12–14^. With the goal of understanding direct neural reprogramming, an initial network hypothesis has been published^13^, but its ability to reproduce and explain the behaviour of the cell has not yet been verified in a data-driven analysis.

In this study, we built a quantitative model of the gene regulatory system governing direct reprogramming to neurons, based on known gene interactions that have been described in literature. We then measured the expression levels of key transcription factors at different time points during a neuronal reprogramming experiment, and used the resulting experimental data to evaluate the literature-based model. We found that this literature-based model was not able to explain the experimentally observed dynamic behaviour, and that the data suggested a missing feedback loop in the gene regulatory network. Accordingly, specific adjustments to the network topology could drastically increase the match between model simulations and experimental observations. In particular, all the adjustments that were shown to substantially improve the model were characterized by an activation from nPTB to PTB, not previously described in literature. Further, we showed that the gene regulatory network equipped with this additional interaction can successfully predict the reactions of the cells to various overexpression and knockdown perturbation experiments, including several different neuronal conversion strategies. In contrast, models based on network hypotheses without this crucial interaction could not reproduce the correct system behaviour. Together, these results suggest that nPTB feeds back into the neural conversion gene network playing an activating role in the expression of PTB, for example by blocking the negative self-regulation of PTB. Thus nPTB induces genes that are opposing neural conversion. In support to this, we experimentally show that a knockdown of nPTB results in successful neuronal conversion. In addition, we present a novel approach to dissect the model to reveal the influence of individual interactions during the conversion process, potentially illuminating internal mechanisms of the network that are hard to observe in the lab. By incorporating the mechanisms of different conversion methods into one explanatory framework, this work aims towards an integrated and predictive understanding of cellular decision making in direct neuronal reprogramming.

## Results

### Measured transcription levels during neuronal reprogramming

**Figure 1:**
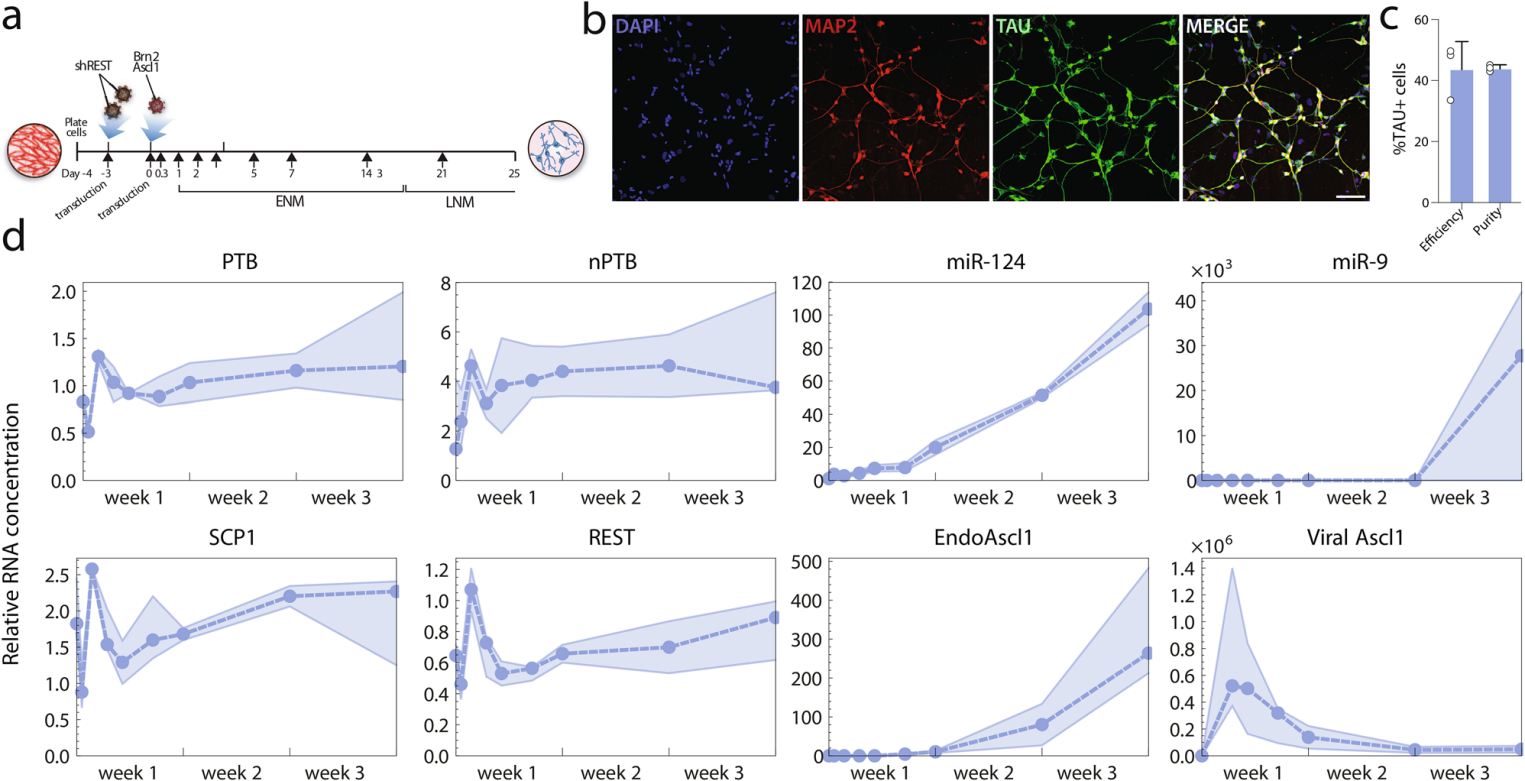
Measured transcription levels during neural reprogramming. (**a**) A schematic representation of the reprogramming process. (**b**) Immunofluorescence staining of MAP2 (in red) and TAU (in green) showing reprogrammed iNs at day 25 post transduction. Cells are counterstained with DAPI (in blue). Scale bar = 100 µm. (**c**) Quantification of TAU + cells at day 25 post transduction, error bars are defined as S.D. (**d**) Measured transcription levels. The dashed line represents the median of three replicates, surrounded by a shaded region between the minimum and maximum values at each time point. The measurements are normalized so that a value of 1 is equal to the fibroblast level, or to the detection limit in the case of Ascl1, which was not detected in fibroblasts. *ENM* early neuronal medium, *LNM* late neuronal medium. The graphs were generated using Graphpad Prism 8, the fluorescent images were put together using Adobe Photoshop 2020, the figures were ensembled using Adobe Illustrator 2020.https://www.graphpad.com/scientific-software/prism/, https://www.adobe.com/se/products/illustrator.html.

We have previously shown that cells that are converted by knocking down REST adopt a transcriptome that more closely resembles that of a neuron, compared to the reprogramming approach involving the overexpression of miR-9/9* and miR-124^6^. However, the combination of both the REST knockdown and miR-9/9* and miR-124 overexpression promotes neuronal maturation^10^. By building on this work, we sought to have a better understanding of how the different components of the gene regulatory network (GRN) controlling direct conversion to neurons interact. We thus generated induced neurons (iNs) through the knockdown of REST, followed by the forced expression of the transcription factors Ascl1 and Brn2 (Fig. 1a). This reprogramming protocol, which leads to a striking change towards a neuronal morphology during the first 25 days of conversion^31^, generates iNs that express synaptic markers such as synapsin, synaptophysin and that are electrophysiologically functional^6^, and genes of specific neurotransmitter phenotypes, including somatostatin (SSTR1), GABAergic (GABRA1), glutamatergic (GRIA2), acetylcholinergic (CHRMA43), and dopaminergic (DRD1)^10^. The neuronal identity of the reprogrammed cells was also confirmed in this study by the expression of the mature neuronal markers TAU and MAP2 at day 25 (Fig. 1b). This reprogramming strategy generates iNs at an efficiency of 43.9 ± 8.9%, as calculated by the percentage of the total number of TAU^+^ cells over the number of starting fibroblasts, as well as a purity of 44.1 ± 1.1%, as calculated by the percentage of the number of TAU^+^ cells over the total number of cells in the dish at day 25 (Fig. 1c). We measured mRNA expression of known key transcription factors in the fibroblast before conversion, as well as three days following the REST knockdown, and 8 h, 1, 2, 3, 5, 7, 14, and 21 days following the induction of viral expression vectors (Fig. 1d). Endogenous Ascl1 and Brn2 were measured separately from their viral counterparts. No endogenous Brn2 transcription was detected.

Our measurements showed intense fluctuations in the expression levels during the first 5 days, followed by stagnation or monotonous increase during the next 2 weeks. This is consistent with previous observations that commitment to neuronal development occurs quickly in the reprogramming process in human cells, followed by a longer period in which downstream genetic and epigenetic control mechanisms are rewired^15–17^. The observed dynamics are typical of a system responding to an initial input shock before settling into a new stable regime. In particular, the dampened fluctuations that are seen in the expression of these genes over the first few days is characteristic of gene regulatory networks containing a negative feedback loop.

We noted that the viral expression was not strongly correlated with the other expression curves, and even at lower observed levels was several orders of magnitude above endogenous expression in fibroblasts and throughout the conversion experiment. In the rest of this study, we assumed that the regulatory mechanisms depending on Ascl1 were saturated during the time frame where it was virally expressed, and considered the absence or presence of Ascl1 as an on–off switch in our models.

### Testing the literature-based network

**Figure 2:**
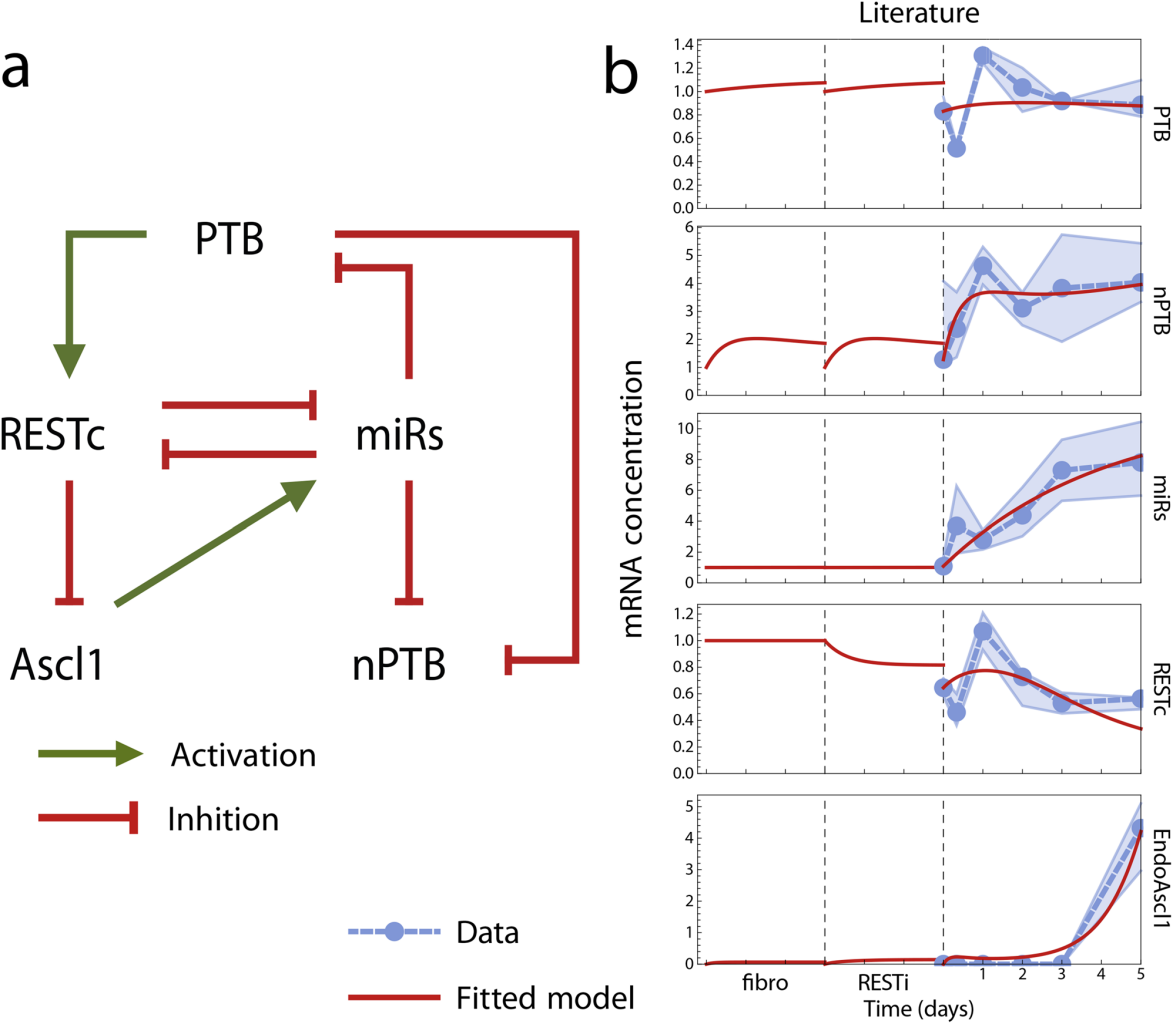
Known interactions are not sufficient to explain observed reprogramming dynamics. (**a**) Literature-based network topology. Green pointed arrows represent activations, red flat arrows represent inhibition interactions. (**b**) Predicted transcription levels according to quantitative model based on gene network in A, fitted to our experimental data. Time scale is divided in three phases, corresponding to fibroblast, REST knockdown, and conversion stages of the experiment. The plots were made with Mathematica 11.3.0 (Linux version). https://www.wolfram.com/mathematica/.

In the last decade, there has been considerable interest in the interaction between several key factors that play an important role in establishing and maintaining neural identity. Based on these studies, we integrated the state of the art for understanding relevant gene interactions (Table 1) into a gene regulatory network model with dynamics suitable for comparison with our dataset (Fig. 2a). This GRN is based especially on previous work by Xue et al.^12,13,19^. We focused here only on neuronal conversion, not maturation.

**Table 1:** Genes in the core regulatory network.

**REST complex** RE1 silencing transcription factor and its cofactors repress many neural genes in non-neural cells^32,33^. Functions as a barrier to neural differentiation, especially in human adults^6^. Knockdown enhances reprogramming efficiency^6,11,28^, but does not cause reprogramming by itself in vitro^34^, while forced constitutive expression prevents successful differentiation^26,35^ Represses the expression of miR-124^6,36,37^ Represses many neuronal genes including Ascl1^37^
miR-124 and miR-9/9* (miRs) Micro-RNAs found in neurons, responsible for repressing a wide array of non-neuronal genes^36^. Overexpression leads to a neuron-like expression profile^17,26^, and conversely, forced downregulation leads to a more non-neuronal profile^36^ Downregulate the activity of the REST complex^28,37,38^ Represse PTB, and to a lesser extend nPTB, during neuronal differentiation^27^
**PTB and nPTB** Polypyrimidine tract binding protein and its neural paralog control alternative splicing (often followed by nonsense degradation^23^) of many neural genes^21^. nPTB replaces PTB during neural differentiation, and its expression decreases as part of neuronal maturation^39^. Knockdown of PTB leads to neural reprogramming^16^ (but not in all cases^22^), while forced expression blocks neuronal differentiation^26^ PTB competes with the repression of SCP by miRs^16^, thus activating REST PTB represses nPTB as well as itself^27^; nPTB has the same functionality^22^
**Ascl1** Most prominent member of a family of proneural basic-helix-loop-helix proteins that control neural differentiation but also proliferation of neural progenitors^40,41^. Overexpression leads to neural conversion^42^ (but not in all cases^26^), especially in combination with other factors^7,29^. Knockout blocks neuronal differentiation in parts of the mouse brain^43^ miR-124 is overexpressed in cells transfected with Ascl1, indicating an activating interaction or pathway from Ascl1 to miRs^29^

These factors have been indicated as key to establishing and maintaining neural identity.

Two nodes in our network represent the combined expression of multiple physical regulatory elements: the miRs node represents miR-124 and miR-9/9* while the REST complex node is an aggregate of its multiple components, including the REST gene and SCP1 cofactor. This approach was motivated by high similarity between the relevant transcription levels during our experimental time frame (correlation coefficient of median concentration levels $r=0.89$ (miR-124–miR-9) and $r=0.89$ (REST–SCP1)), especially during the first five days ($r=0.97$ (miR-124–miR-9) and $r=0.93$ (REST–SCP1)). Thus, we observed almost no quantitative distinction between these regulatory elements during the conversion process. Experimental data gathered from other conversion methods could potentially distinguish the action of these factors in the future.

We developed a quantitative computational model for the GRN in Fig. 2a, using the Shea-Ackers formalism^20^, yielding a system of ordinary differential equations that predicted the dynamics of RNA concentrations over time for each node in the network. The interaction strengths and reaction rates were unknown parameters in these equations; we used an evolutionary algorithm to find the parameter values which yielded the closest match between predicted and observed transcription dynamics. The model was executed in three modes corresponding to the three stages of our experimental conversion process: the initial fibroblast cell stage, the REST knockdown stage, and the conversion stage with Ascl1 and Brn2 overexpression. The switch between the three modes was modelled by manipulating two parameters representing the action of external factors i.e. REST knockdown and Ascl1 overexpression, respectively. System parameters were optimized to maintain a steady state in the fibroblast stage, to match the observed expression after 3 days of REST inhibition, and to fit as closely as possible the expression levels dynamics during the conversion stage after viral activation. The optimisation and modelling procedures are explained in more detail in the "Methods" section.

When fitting our simulation outcomes to the experimental time series data, we found that our initial model based on interactions described in literature was not able to explain the dynamic behaviour of the system (Fig. 2b). The following observations stood out as possible explanations for the inability of the model to fit the data.

While the behaviour of the system during the first five days of observation appeared to be dominated by the shock response to the sudden addition of the viral vector-delivered Ascl1 to the system, there was no interaction in the network that could explain these heavy fluctuations in expression levels. In particular, the strongest fluctuations occurred during the first day, when PTB, REST, and miRs drastically switched from increasing to decreasing transcription levels, or vice versa. These fluctuations are evidence of a negative feedback loop in the network: a gene that activates its own repressor, or represses its own activator, causes a delayed self-repression that can result in several rounds of increase and decrease of transcription after sudden changes. No negative feedback has been suggested before in literature on direct neural reprogramming dynamics.

In the literature based GRN, Ascl1 was controlled exclusively by inhibition from the REST complex. Therefore, it was activated only by constitutive transcription factors not present in the network. However, REST reaches its lowest expression value during the first day, where little or no Ascl1 is expressed (Fig. 1d). This suggested that another regulatory process might have been necessary to accurately explain why Ascl1 is expressed after five days but not before. Alternatively, it is possible that the low REST expression is simply not sustained for long enough during the first day to accumulate sufficient Ascl1 protein concentration.

Finally, we note that previous publications have described inhibiting interactions between PTB and nPTB, as both proteins have been found to inhibit the correct splicing of their own and each other’s mRNA transcripts^21–23^. Of these interactions, only the inhibition of nPTB by PTB was included in the literature model. Self-inhibition and regulation of PTB by nPTB have not received attention in literature as causal elements influencing the reprogramming process, and have not been included in previous models that integrate important interactions into a GRN^12,13,19^ (Fig. 2a). However, it has not been ruled out that these interactions play a role in the quantitative expression dynamics of the neural reprogramming system.

### Comparison of alternative network topologies

**Figure 3:**
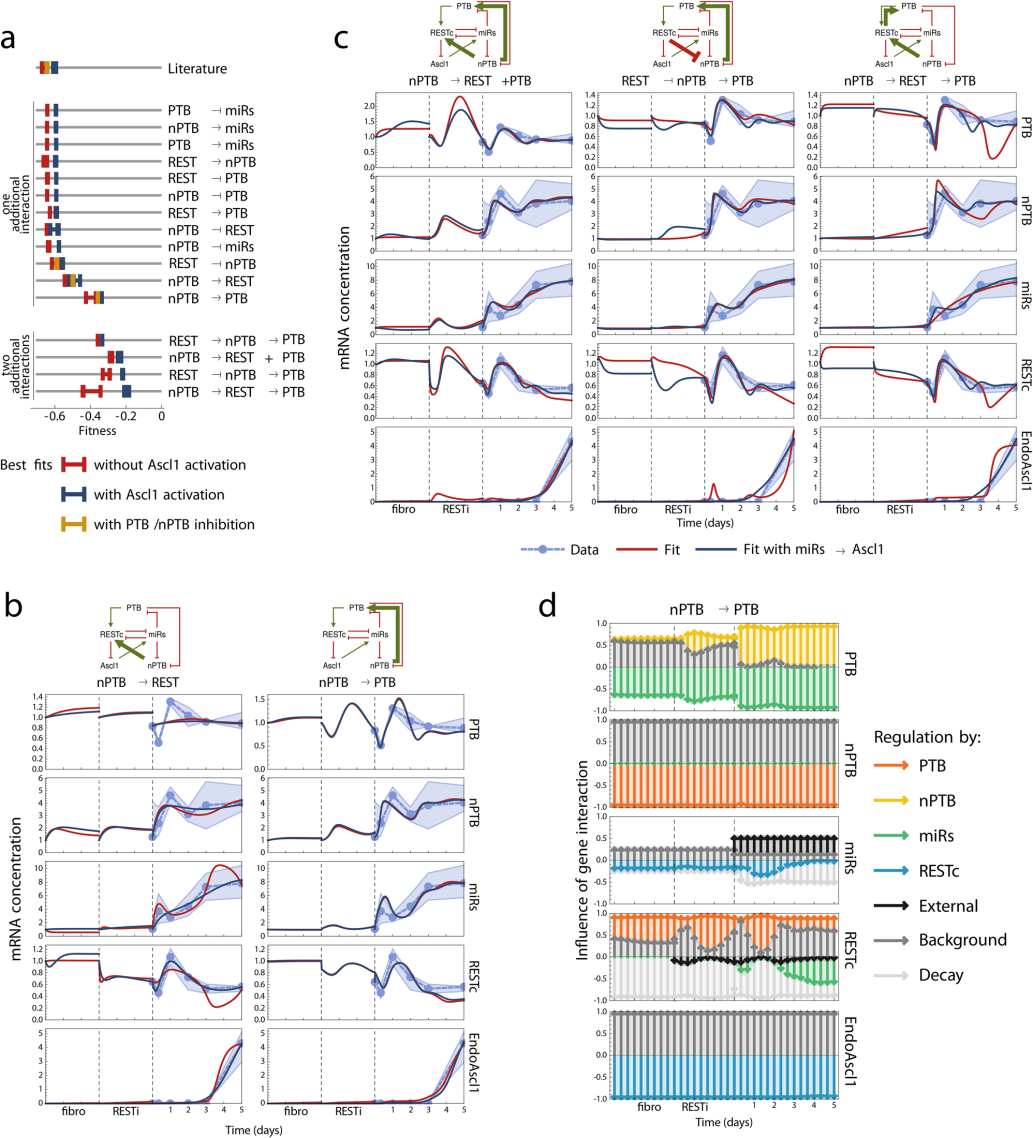
Model performance increases drastically when nPTB activates PTB. (**a**) Model performance as measured by the objective (fitness) function of the optimization procedure, that is, the negative fitting error. Performance of the three best fits is shown. Models are grouped by the hypothetical interaction they have in addition to the literature-based network, where $\to$ denotes activation, and ${ \dashv }$ denotes inhibition. (**b**) The time evolution of the RNA concentrations predicted by the nPTB $\to$ REST and nPTB $\to$ PTB models. Time scale is divided in the three experimental phases as in Fig. 2b. (**c**) The time evolution of the DNA concentrations in the best models with two additional interactions. (**d**) Dissection of the simulated time evolution in the nPTB $\to$ PTB model (see (**b**)), decomposing the transcription rates of each factor into the separate contributions of its activators and inhibitors. Activating interactions are represented by arrows pointing upwards from the zero line, inhibiting interactions point downwards. Arrow length indicates relative influence of the regulator. See Methods for an extended description. nPTB and Ascl1 are controlled by changes in inhibition regulation that are too small to see on the figure. The plots were made with Mathematica 11.3.0 (Linux version). https://www.wolfram.com/mathematica.

To investigate whether a small modification to the GRN topology could improve the match between predicted and observed transcription levels, we attempted to fit models with alternative sets of gene interactions to our data. When adding a new hypothetical interaction to the network, a quantitative model was constructed and trained in the same systematic way as the original literature-based model. Then fitting errors were compared (Fig. 3a).

We hypothesised that a missing control mechanism for Ascl1 is responsible for the lack of fit between the literature model and measurements, as discussed in the previous section. In terms of model fitting, this hypothesis entails that RESTc is constrained to remain at higher expression levels during the first day in order to avoid activation of Ascl1, and that this constraint potentially affects the regulation of other genes by RESTc. To test whether the ability of the model to fit observed data is indeed constrained in this way, we constructed an alternative model where Ascl1 was activated at the correct time, so that RESTc is no longer constrained by the need to regulate Ascl1. Because miRs reach maximal expression at the same time as Ascl1 in our data, it was convenient to implement this in practice as an activation of Ascl1 by miRs, which allows Ascl1 to be activated at the correct time with an appropriate set of parameters without constraining RESTc or miRs. Figure 3a (blue) shows that introducing an activation of Ascl1 by miRs resulted in a small improvement compared to the unmodified literature model.

We also considered the potential role of self-inhibition of nPTB and PTB, as well as inhibition of PTB by nPTB, which are interactions that have been observed experimentally. The fitting errors of the resulting model are shown in Fig. 3a (yellow). The PTB and nPTB inhibitions did not affect the ability of the model to match observed data.

We further investigated the hypothesis that another, previously unknown interaction is missing in the GRN. To this end, we constructed models for all possible network alternatives where a single interaction was added to the initial literature-based network. The added interactions were between the four active nodes PTB, nPTB, RESTc and miRs, excluding self-interactions (a node activating or repressing itself) and double-interactions (a node acting as an activator and repressor for the same target). The fitting error of these models is shown in Fig. 3a (one additional interaction).

For each of the tested models, there is also an alternative where Ascl1 is activated by miRs, increasing the freedom to fit RESTc (shown in blue); and another alternative where self-inhibition of PTB and nPTB, and inhibition of PTB by nPTB, were included (shown in yellow). We note that this comparison includes many different models, and therefore introduces the potential dangers associated with multiple hypothesis testing. However, comparison between multiple similar models is also a useful indicator of whether any fitting improvements are unique to a particular hypothesis, or simply result from the generic increase in free parameters.

Adding new interactions (and thus more free parameters) did not affect the fitting error of these models in most cases. Only two models showed a marked improvement, and in particular the match between simulated and observed gene expression was enhanced when nPTB activated PTB (nPTB $\to$ PTB). To a lesser extent, fitting also improved when nPTB activated REST (nPTB $\to$ REST). The expression levels predicted by these models over the course of the conversion experiment are shown in Fig. 3b. Notably, the nPTB $\to$ PTB model introduces direct negative feedback between nPTB and PTB, a network motif that was strongly suggested by visual inspection of the data. The two models (nPTB $\to$ PTB and nPTB $\to$ REST) are similar in the sense that each implies the other through an intermediate reaction chain (nPTB $\to$ [PTB $\to$] REST and nPTB $\to$ [REST ⊣ miRs ⊣] PTB).

To further investigate these interactions, we questioned whether an even better fit to our data could be obtained by combining two different interactions (not including the additional activation of Ascl1 by miRs). We constructed several models with two interactions between PTB, nPTB and RESTc. These combinations of multiple interactions resulted in a small decrease of the fitting error in some cases, more so when RESTc was not constrained to the regulation of Ascl1, as shown in Fig. 3a (two additional interactions). However, on visual inspection, there is no substantial change in the fit between the simulated and observed expression dynamics, despite the added free parameters, as shown in Fig. 3c. These experiments indicate that the improvement obtained by adding the nPTB $\to$ RESTc interaction are subsumed by the nPTB $\to$ PTB model, since combining both interactions (labelled nPTB $\to$ PTB $+$ RESTc) did not result in a marked improvement compared to the simpler nPTB $\to$ PTB model. Thus, we can consider the nPTB $\to$ RESTc model a weaker version of nPTB $\to$ PTB.

These results single out nPTB $\to$ PTB as the most valuable hypothetical interaction with respect to improving the fitting error of our models. Two other interactions could decrease fitting error (nPTB $\to$ RESTc and miRs $\to$ Ascl1), but when combined with nPTB $\to$ PTB these interactions are both shown to be obsolete. Indeed, the nPTB $\to$ PTB model could not be improved by the added freedom in RESTc conferred by the miRs $\to$ Ascl1 interaction (Fig. 3b), unlike all other models (Fig. 3a). Together, these results show that an activating interaction between nPTB and PTB was highly compatible with our data.

We finish this section with a more detailed view of the nPTB $\to$ PTB model. The equations governing the quantitative models presented here were built up from the described gene interactions using a systematic procedure. Different terms in these equations correspond to specific gene interactions. By comparing the magnitude of partial expressions that represent contributions of individual gene interaction, we can measure and visualize the relative influence of each activator or inhibitor on the total transcription rate of each gene at different time points during a simulation. This dissection of the model allows us to distinguish the roles of different gene interactions by directly observing the (model) system’s internal mechanisms in full detail. This is a unique benefit of computational modelling, since in the lab it is only possible to observe the behaviour of the network as a whole, for example in response to overexpression or knockdown of genes, leaving the contributions of individual interactions to inexact inference. Figure 3d shows such a dissection analysis for a simulation of the nPTB $\to$ PTB model (the corresponding timed expression profile is shown in Fig. 3b). See Methods for the analytical details of this analysis. Figure 3d shows that the inhibition from miRs has a negligible impact on nPTB expression in the model after training on the observed data. This interaction may still contribute to the behaviour of the real cell, but apparently its function is redundant (i.e. is functionally equivalent to the inhibition by PTB) in the conditions of our conversion strategy.

### Model performance in overexpression and knockdown scenarios

Having compared several network hypotheses that are highly compatible with gene expression data during one conversion process, we set out to investigate whether the fitted models would also reproduce the behaviour of the cell in response to other stimuli. To this end, each model was subjected to simulated overexpression (OX) or knockdown (KD) of factors that are known to cause or block neural conversion (see Table 1). The results could then be compared to experimental observations. Since these qualitative data are independent of our training data, they are suitable for model testing and validating comparison.

In order to simulate analogues to these experiments in our models, we explored the effect of modifying relevant parameters on the system behaviour. Viral induction of Ascl1 and REST knockdown were explicitly represented in the system equations and thus could be controlled by manipulating the dedicated system parameters. The same method was used when we simulated the switch between the three stages of the experimental procedure. All other cases of overexpression and knockdown were simulated by increasing (5×) or decreasing (1/5×) the transcription rate coefficient of the factor under consideration. The resulting system of equations were then integrated to yield the evolution of transcription levels over time, starting from the fibroblast mRNA expression level as an initial state. Because many direct reprogramming methods operate at low efficiency, the deterministic integration representing bulk average expression levels of a population was supplemented by a stochastic approach, to capture noise in individual cells. We used the Gillespie algorithm to produce stochastic simulations over a simulated time span of 14 days^25^ which introduces gene regulatory network intrinsic noise in the system. Endogenous expression of Ascl1 was taken as a convenient indication that a simulated cell had committed to a neuronal fate; a maximum endogenous Ascl1 value of 1 is taken as the threshold value to indicate conversion. Results are shown in Table 2 and Supplementary Fig. 1 for the models with the lowest fitting error (alternative models with miRs $\to$ Ascl1 or PTB/nPTB inhibition are not shown).

**Table 2:** The nPTB $\to$ PTB model correctly predicts conversion outcome of most overexpression and knockdown scenarios.

		Fibroblast	OX Ascl1	OX miRs	KD REST	OX Ascl1 + KD REST	OX Ascl1 + OX REST	OX Ascl1 + OX PTB	KD PTB
Simulation outcome	**Lab outcome**	**No**	**Yes**	**Yes**	**No**	**Yes+**	**No**	**No**	**Yes**
	Literature model	**No**	**Yes**	**Yes**	**No**	Yes	Yes	Yes	No
	nPTB $\to$ REST	**No**	**Yes**	**Yes**	Yes	Yes	**No**	Yes	No
	**nPTB** $\to$ **PTB**	**No**	**Yes**	**Yes**	**No**	**Yes+**	**No**	Yes	No
	nPTB $\to$ REST $+$ PTB	Yes	**Yes**	**Yes**	Yes	Yes	Yes	Yes	No
	nPTB $\to$ REST $\to$ PTB	**No**	No	No	Yes	**Yes+**	**No**	**No**	No
	REST ${ \dashv }$ nPTB $\to$ PTB	Yes	**Yes**	**Yes**	Yes	Yes	**No**	**No**	**Yes**

Conversion outcome is shown for several OX and KD experiments described in literature (discussed in Table 1), and compared to outcome predicted by quantitative models. Simulated outcome was considered to predict conversion (“Yes”) if Ascl1 expression exceeded 1 at any time point during at least 1 out of 50 simulations, and no conversion (“No”) otherwise. In the case of OX Ascl1 + KD REST, all models predict conversion because it is an explicit feature of the training data. For this reason, a higher conversion efficiency was required (indicated by a plus sign) in this experiment as compared to OX Ascl1. Bold cells indicate outcomes that correspond to lab experiments. Full simulation results are shown in Supplementary Fig. 1.

**Figure 4:**
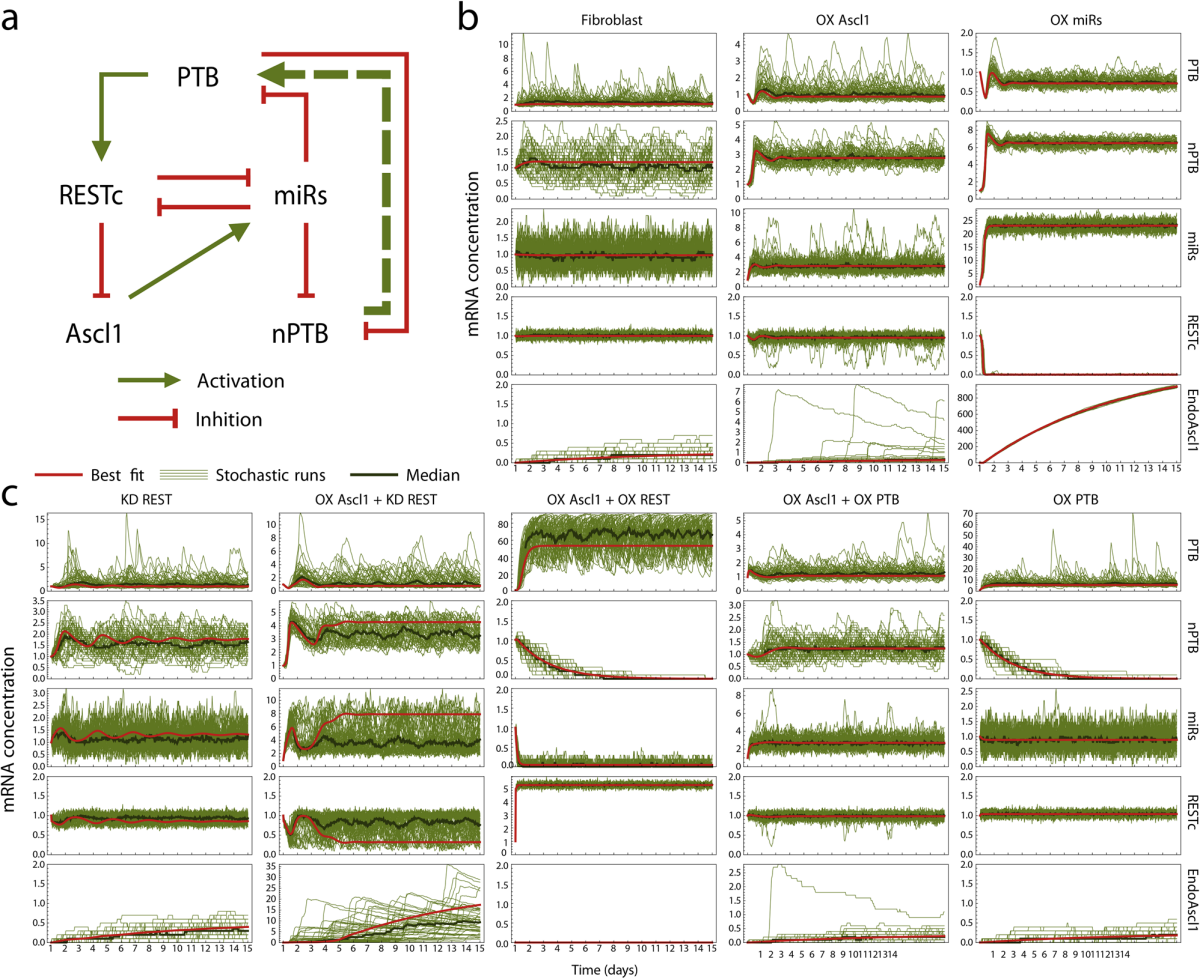
The nPTB $\to$ PTB model correctly predicts conversion outcome of most overexpression and knockdown experiments. (**a**) Network topology of the nPTB $\to$ PTB model. (**b**,**c**) The deterministic (red) and stochastic (green) simulation outcomes are shown for OX/KD scenarios in the nPTB $\to$ PTB model. Similar simulations for all models and experiments are summarized in Table 2 and are shown in Supplementary Fig.1. The plots were made with Mathematica 11.3.0 (Linux version). https://www.wolfram.com/mathematica/.

Out of all the models presented here, nPTB $\to$ PTB (Fig. 4a) provided the most accurate predictions of system behaviour in different OX/KD scenarios, improving substantially upon the purely literature-based model. The stochastic and deterministic simulation results corresponding to OX/KD scenarios with the nPTB $\to$ PTB model are shown in Fig. 4b,c.

Stochasticity of these simulations is necessary to capture heterogeneity of cellular populations. In particular, overexpression of Ascl1 only caused neuronal conversion (endogenous Ascl1 expression) in some simulations, and the conversion efficiency (fraction of simulations with high endogenous Ascl1 levels) could be greatly increased by addition of REST knockdown, in the nPTB $\to$ PTB model. This is in accordance with experimental results^6^. However, it bears mentioning that simulated experiments with Ascl1 overexpression correspond most closely to the conditions of the training data for these models. In other conditions, simulated heterogeneity is less realistic, for example in the case of miRs overexpression. In general, single-cell data would be required to make quantitative predictions of conversion efficiencies.

### Experimentally validating the nPTB $\to$ PTB model

**Figure 5:**
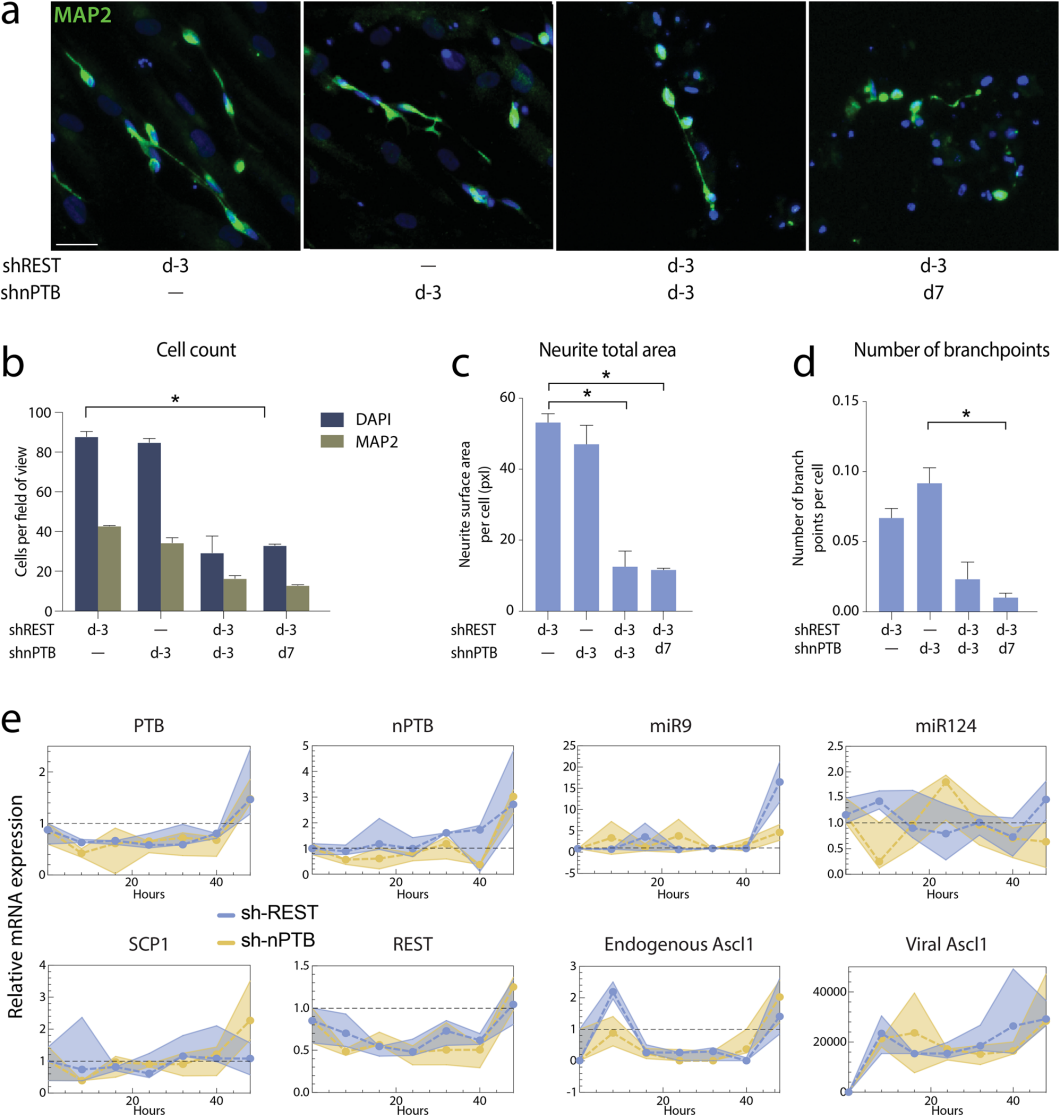
Experimental validation of the nPTB $\to$ PTB model. (**a**) Immunofluorescence staining of MAP2 (in green) showing reprogrammed iNs at day 25 post transduction. Cells are counterstained with DAPI (in blue). Scale bar = 150 µm. (**b**) Quantification of DAPI + and MAP2 + cells at day 25 post transduction, error bars are defined as S.E.M. (**c**,**d**) Quantification of neurite total area (**c**) and number of branch points, error bars are defined as S.E.M. (**d**) in iNs at day 25 post transduction. (**e**) Measured transcription levels. The dashed line represents the median of three replicates, surrounded by a shaded region between the minimum and maximum values at each time point. The measurements are normalized so that a value of 1 is equal to the fibroblast level, or to the detection limit in the case of Ascl1, which was not detected in fibroblasts. The graphs were generated using Graphpad Prism 8, the fluorescent images were put together using Adobe Photoshop 2020, the figures were ensembled using Adobe Illustrator 2020. https://www.graphpad.com/scientific-software/prism/, https://www.adobe.com/se/products/illustrator.html.

In order to test the existence of feed-back from nPTB to the core neural conversion network by inducing PTB and subsequently opposing the neural conversion, we conducted a nPTB knockdown experiment. Our results showed that a nPTB knockdown generates iNs at a similar efficiency to the one obtained through the repression of REST (Fig. 5a,b). Furthermore, the complexity of the neuronal morphology, as assessed by measurements of the total surface area of neurites and the number of branchpoints, was also similar, suggesting that both strategies are equipotent (Fig. 5c,d). Thus, our results showed that repression of PTB by itself and nPTB does not improve neuronal conversion while a knockdown of nPTB, which is a PTB inducer, leads to iNs conversion. We also observed that simultaneous depletion of REST and nPTB during the initial phase of conversion results in a lower conversion efficiency and neuronal complexity (Fig. 5c,d). We speculate here that this double depletion leads to expression of REST which is under the levels necessary for successful conversion to iNs.

To test whether the nPTB activation is crucial at the initial conversion stage but not required for the neuronal maturation, we have tested experimentally whether a nPTB knockdown done after the initiation stage would improve maturation. We have previously identified that the major changes in gene expression occur within the first 5 days after the initiation of conversion^6,10^. We thus initiated the nPTB knockdown on day 7 at the beginning of the maturation phase, which did not improve on the number of neurons, and significantly gave rise to less complex neuronal morphology (Fig. 5c,d). This suggests that nPTB is important for the maturation of the neurons when the conversion is initiated through a depletion of REST.

Finally, to compare between the REST KD- and the nPTB KD-based reprogramming, we have performed gene expression profiling of the main actors in our model at 8hrs intervals during the first 48 h post transduction with Ascl1 and Brn2. Please note that through this experiment we also performed a zoom in exercise by monitoring the gene expression more often than in our training data. Our results show that PTB, REST and endogenous Ascl1 follow the same transcriptional fluctuations during the first 48 h during both the REST KD and the nPTB KD experiments. The resulting experimental time series data showed PTB, REST and endogenous Ascl2 expression downregulation early on followed by an increase around the second day of reprogramming (Fig. 5e), suggesting the presence of a negative feedback loop in the network in both KD cases. Furthermore, the knockdown of nPTB results in a slight downregulation of PTB expression confirming our model prediction of positive interactions between nPTB and PTB. We also observed that nPTB KD lead to diminished expression of REST which is in line with the double positive input on REST from nPTB via PTB. However, the REST knockdown did not impact on nPTB expression levels, suggesting that the double negative interaction between REST and nPTB via miRs is not a crucial mechanism in the network. These results suggest that the successful neuronal conversion seen in (Fig. 5a,b) could be the result of the REST downregulation in these cells either directly or through depletion of nPTB which positively induces REST through PTB.

### Dissecting the model response to PTB knockdown

Generally, there was a remarkable agreement between the system behaviour predicted by the nPTB $\to$ PTB model and experimental observations, even though the training data cover only a narrow set of circumstances compared to the many perturbation experiments used as validation. The fact that a computational model could learn the outcome of one experiment from training data based exclusively on another experiment suggests that they operate in the same region of the system phase space, and that these different conversion strategies invoke a comparable response from the GRN. Notably, the exception to this rule is the system response to PTB knockdown (which can cause neuronal differentiation^16^) or overexpression (which is known to block neuronal differentiation with Ascl1 overexpression^26^). This suggests that neuronal conversion induced by PTB knockdown is affected by a different cascade of gene interactions compared to the other conversion strategies. Based on the gene interaction network, one way in which the PTB knockdown strategy stands out is that it affects REST (and through it the rest of the network) by removing a crucial activator (PTB), whereas other conversion strategies directly or indirectly induce miR-124-9/9*, a repressor of the REST complex.

**Figure 6:**
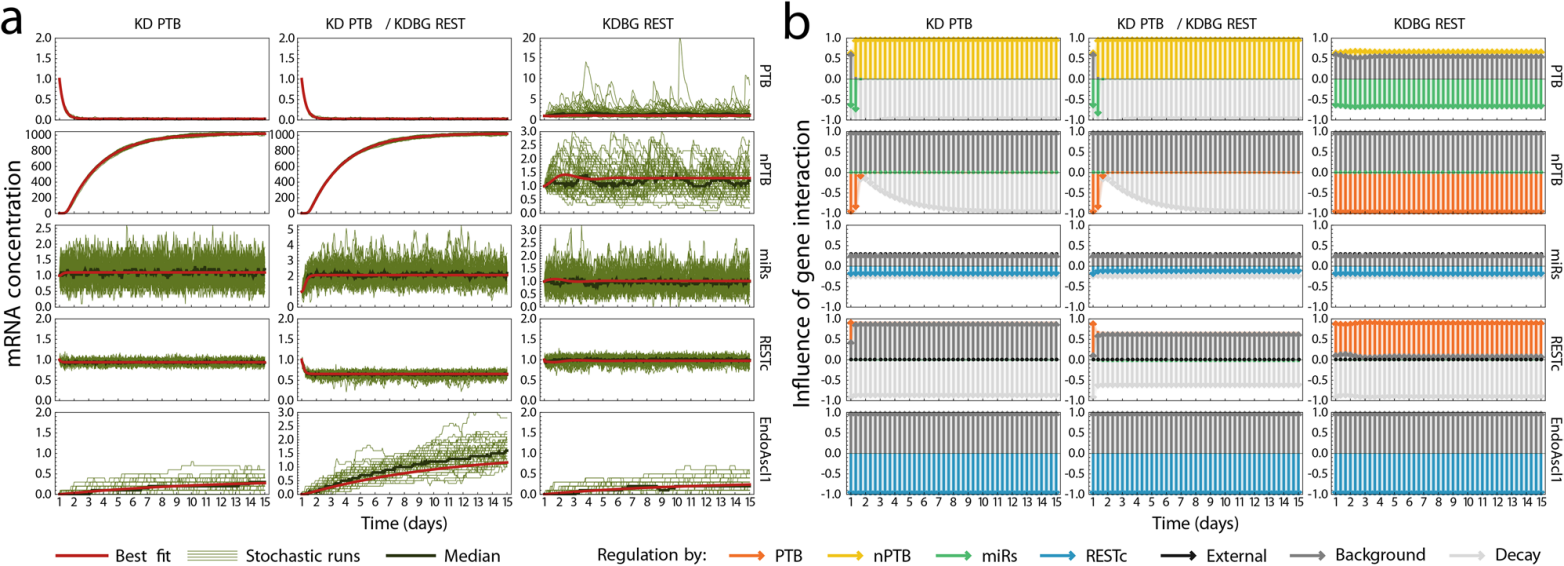
Strong constitutive activation of REST expression blocks neuronal reprogramming through PTB knockdown in models. (**a**) Stochastic (green) and deterministic (red) time evolution of the system. Left: simulated knockdown of PTB. The system remains in non-neural state, as seen by the lack of Ascl1 expression. Middle: combining PTB knockdown with a diminished constitutive REST activation induces Ascl1 expression. Right: with regular PTB expression, reducing constitutive REST activation results in a stable non-neuronal profile, with a steady state that strongly resembles baseline fibroblast levels. (**b**) The influence of different regulatory interactions on the expression of each factor during the deterministic experiments shown in panel (**a**) (see also Fig. 3d). See Methods for an extended description. The plots were made with Mathematica 11.3.0 (Linux version). https://www.wolfram.com/mathematica/.

We sought to understand this in more detail by applying the model dissection shown in Fig. 3d to relevant overexpression and knockdown scenarios for the nPTB $\to$ PTB model. Simulated PTB knockdown affected the model by an increase of nPTB, but with no noticeable change in the expression of other factors (Fig. 6a, left). While different from the expectation that PTB knockdown results in neuronal conversion (Table 1), this outcome has also been found experimentally^22^. In our model, this diminished system response is caused by the fact that RESTc expression remained activated constitutionally, maintaining nominal concentrations, even when RESTc was no longer activated by PTB (Fig. 6b, left). Thus, PTB knockdown does not affect RESTc concentrations and therefore its effects do not cascade through the rest of the GRN. Interestingly, when the constitutive activation of REST was reduced, the system became permissible to neuronal conversion by PTB knockdown (Fig. 6a,b, middle). In the absence of PTB knockdown, this lower constitutive RESTc expression did display different system behaviour compared to the unmodified parameter set (Fig. 6a,b, right). Moreover, our experimental data in Fig. 5 showed that nPTB KD results in neural conversion therefore an nPTB KD could lead to depletion of the constitutive REST expression. Thus, the constitutive RESTc activation is largely a free parameter when fitting to our data. Note that constitutive here refers to any regulation by factors that are not explicitly present in our GRN. It does not imply that this regulation is identical for every cell, or independent of the cell’s state and environment outside the range of our experiments. These results show that our data and model predict that some cells are more susceptible to neuronal conversion through PTB knockdown than others, due to variation in constitutive expression of members of the REST complex.

## Discussion

In this study, we put forward a quantitative model of the core GRN governing direct reprogramming of human adult fibroblasts to neurons. Our analysis showed that a straightforward interpretation of the known interactions between key transcription factors was unable to explain the full dynamic behaviour that we observed. In particular, transcription data gathered during a conversion experiment showed evidence of a negative feedback loop in the GRN, which has not previously been described.

Adding a hypothetical interaction to the network where nPTB activates PTB introduced such a negative feedback loop and drastically increased the match between measured mRNA expression levels and those predicted by the model. In addition, in contrast to various alternative hypotheses, this model correctly predicted the response of the cell in a range of different overexpression and knockdown scenarios. Taken together, these findings suggest that such an interaction between these two genes may play a role in neuronal conversion. In support to this, we show that modulating this interaction through a nPTB repression results into successful neuronal reprogramming. However, the possibilities for how this interaction is manifested biochemically should be interpreted broadly. The model is agnostic of specific regulatory mechanisms, so the cause of the predicted interaction is not necessarily restricted to the transcriptional level and may include mediation by one or more intermediate regulators.

Interestingly, there is a possible mechanism by which nPTB may function as an activator of PTB, in line with known features of PTB and nPTB regulation. Both PTB and nPTB are known to regulate the splicing of PTB mRNA, effectively inhibiting its expression by inducing nonsense-mediated decay^21–23,27^. Furthermore, it has been shown that PTB and nPTB have similar RNA-binding properties, however, nPTB is a weaker splicing regulator than PTB due to a difference in recruitment of cofactors^21,22,27^. Therefore, whether the net effect of nPTB on the correct splicing of PTB is positive or negative depends on the present concentration of PTB. If the concentration of PTB is low, nPTB simply acts as a splicing regulator and decreases PTB expression. On the other hand, if the concentration of PTB is high, binding of nPTB to the PTB mRNA is likely to displace PTB as a splicing regulator, and cause a net increase in correctly spliced RNA due to its relatively weaker activity. It is possible that this mechanism explains our findings, wherein nPTB is best modelled and understood as an activator (that is, increasing the expression) of PTB in the relevant cellular conditions. Interpreted this way, our computational analysis suggests that known interactions between key regulators are enough to explain much of the cellular decision-making process during direct neuronal reprogramming, although competition between PTB and nPTB for the regulation of PTB plays a more important role than expected. Note that our quantitative approach does not distinguish between weaker or stronger regulators with the same binding strength, so that explicitly including repression of PTB by itself and nPTB did not improve our models.

When investigating the response of the different models to simulated overexpression and knockdown experiments, we found a remarkable ability to predict experimental outcomes. The fact that information gained from only one experiment generalises to other conversion strategies suggests that the different methods operate via the same interaction mechanisms and guide the cell to similar trajectories in the phase space of the system. One consequence of this insight is that we may expect greater conversion efficiencies when compatible strategies are combined. Indeed, several publications have combined REST knockdown, Ascl1 overexpression and miR-124-9/9* overexpression, each of which can induce conversion on its own, with the effect of increasing conversion efficiency and neuronal maturation^6,10,11,28,29^. On the other hand, lower conversion efficiencies can be observed when incompatible strategies are combined, as we show in this study with the double repression of REST and nPTB.

Our analysis did not predict that the conversion strategy based on PTB knockdown would result in cells with a neuron-like identity, but instead showed an increase in nPTB expression without affecting other genes. Interestingly, although PTB knockdown has been used successfully in a number of conversion experiments^16,30^, this negative experimental outcome has also been observed in the lab^22^. We provide a speculative but plausible explanation for this variation in results by observing that, in our best model, the conversion was blocked by constitutive activation of REST. According to the model, cells with a lower constitutive REST activation would have similar REST expression levels, but would be more susceptible to conversion after PTB depletion. We also showed experimentally that nPTB KD leads to successful production of iNs which confirms that nPTB feeds back in the core gene regulatory network controlling direct neural conversion. Moreover, nPTB KD reduced the amount of REST in the cells suggesting that diminished nPTB might be able to block the constitutive REST activation.

Overall, this work illustrates that the current understanding of the genes and interactions governing neural reprogramming can be unified into a computational framework that explains much of the observed behaviour of the cell. Our analysis brings a new perspective to some aspects of the cellular decision process that establishes neuronal fate, and demonstrates a novel analytical tool to dissect internal model dynamics. In the future, more detailed quantitative models informed by data from varied experiments can increase the power of this approach, and single-cell data would improve the ability to make predictions regarding heterogeneity and conversion efficiency. This and future models will help to provide a better understanding of the existing conversion strategies, and suggest improvements that could result in higher yield or faster conversion to neurons.

## Methods

### Experimental data

#### Cell culture and cell lines

Adult dermal fibroblasts of a 61-, 67- and 75-year-old healthy female donors were obtained with written, informed consent from the Parkinson’s Disease Research clinic at the John van Geest Centre for Brain Repair (Cambridge, UK). All experimental protocols were approved by the East of Cambridgeshire Research Ethics Committee (REC 09/H0311/88) as well as the University of Montreal *Comité d’éthique de la recherche en sciences et en santé* (CERSES-18-004-D). All experiments were performed in accordance with the Declaration of Helsinki.

For details on the skin biopsy sampling method, please refer to^6^. Primary fibroblasts were expanded and cultured at 37 °C in 5% CO_2_ in fibroblast medium (Dulbecco’s Modified Eagle Medium (DMEM) + Glutamax (Gibco) with 100 mg/mL penicillin/streptomycin (Sigma), and 10% FBS (Biosera)). The cells were then dissociated with 0.05% trypsin, spun, and frozen in 50/50 DMEM/FBS with 10% DMSO (Sigma).

#### Viral vectors and virus transduction

DNA plasmids expressing mouse open reading frames (ORFs) for Ascl1 and Brn2 on the same construct (pB.pA, see Ref.^6^) in a third-generation lentiviral vector containing a non-regulated ubiquitous phosphoglycerate kinase (PGK) promoter were used (Fig. 1a). The knockdown of REST and nPTB were done using short hairpin RNAs^6,31^. The shRNAs plasmid DNA for nPTB was purchased from Sigma-Aldrich. All the constructs have been verified by sequencing. Lentiviral vectors were produced and titrated as previously described^6^. Transduction was performed at a MOI of 10 for the pB.pA vector and at an MOI of 5 for the shRNA vectors against REST. 0.5 µl of the sh-nPTB lentiviral vector was added to the cells, which provided a 50% downregulation on day 3 (Supplementary Fig. 2).

#### Neuronal reprogramming

For direct neuronal reprogramming, fibroblasts were plated at a density of 26,300 cells per cm^2^ in 24-well plates (Nunc) coated with 0.1% gelatin (Sigma). On the next day, cells were transduced with shRNAs against REST or nPTB and the medium was changed the following day. Three days after this viral transduction, cells were transduced with the pB.pA vector. Fibroblast medium was replaced by neural differentiation medium (NDiff227; Takara-Clontech) supplemented with growth factors at the following concentrations: LM-22A4 (2 µM, R&D Systems), GDNF (2 ng/mL, R&D Systems), NT3 (10 ng/mL, R&D Systems) and the small molecules CHIR99021 (2 µM, Axon), SB-431542 (10 µM, Axon), noggin (0.5 µg/mL, R&D Systems), LDN-193189 (0.5 µM, Axon), as well as valproic acid sodium salt (VPA; 1 mM, Merck Millipore) and db-cAMP (0.5 mM, Sigma), 3 days after the second transduction. Half of the neuronal conversion medium was replaced every 2–3 days. 18 days post-transduction, the small molecules were stopped, and the neuronal medium was supplemented with only the factors LM-22A4, GDNF, NT3 and db-cAMP until the end of the experiment.

#### qRT-PCR analysis

Total RNA, including miRNA, was extracted from cells from the same line at different stages of conversion (Fig. 1a) using the micro miRNeasy kit (Qiagen) followed by Universal cDNA synthesis kit (Fermentas, for RNA analysis; Exiqon or a miRCURY LNA kit for miRNA expression). Three reference genes were used for each qPCR analysis (ACTB, GAPDH and YWHAZ). Primer sequences can be found in Supplementary Table 1. LNA-PCR primer sets, specific for hsa-miR-9-5p, hsa-miR-124-3p and hsa-miR-103 (the latter used as normalization miRNA), were purchased from Exiqon and used for the miRNA qPCR analysis. All primers were used together with LightCycler 480 SYBR Green I Master (Roche). Standard procedures of qRT-PCR were used, and data were quantified using the ΔΔCt-method. Analyses were performed in triplicates at each time point.

#### Immunocytochemistry, quantification, and imaging

Cells were fixed on day 25 in 4% paraformaldehyde, permeabilized with 0.1% Triton-X-100 in 0.1 M PBS for 10 min. Thereafter, cells were blocked for 30 min in a solution containing 5% normal serum in 0.1 M PBS. The following primary antibodies were diluted in the blocking solution and applied overnight at 4 °C: mouse anti-TAU clone HT7 (1:500, Thermo Scientific), chicken anti-MAP2 (1:10,000, Abcam). Fluorophore-conjugated secondary antibody (Jackson ImmunoResearch Laboratories) was diluted in blocking solution and applied for 2 h. Cells were counterstained with DAPI for 15 min followed by three washes in PBS. The total number of DAPI^+^, TAU^+^ and MAP2^+^ cells per well were quantified and imaged using the Cellomics Array Scan (Array Scan VTI, Thermo Fischer), which is an automated process ensuring unbiased measurements between groups. Applying the programs “Target Activation” and “Neuronal profiling”, 289 fields (10X magnification) were acquired in a spiral fashion starting from the center.

### Building quantitative models from GRNs

#### The Shea-Ackers formalism

The Shea-Ackers formalism is a mathematical model that describes the activity of a regulatory sequence as a function of the concentrations of activating and inhibiting factors^20^. Assuming that the regulated gene is active when the binding sequence is occupied by an activator ($\in$ $act$), and inactive when it is unoccupied or in the presence of an inhibitor ($\in$ $inh$), the formalism borrows techniques from statistical mechanics to compute the average activity at the site assuming thermodynamic equilibrium:

1 $$SA\left( X \right) = \frac{{\mathop \sum \limits_{T \in act} \left( {\frac{\left[ T \right]}{{k_{T,X} }}} \right)^{{h_{T,X} }} }}{{1 + \mathop \sum \limits_{T \in act} \left( {\frac{\left[ T \right]}{{k_{T,X} }}} \right)^{{h_{T,X} }} + \mathop \sum \limits_{T \in inh} \left( {\frac{\left[ T \right]}{{k_{T,X} }}} \right)^{{h_{T,X} }} }},$$

where $k_{T,X}$ are dissociation constants describing the interaction strength between factor $T$ and the regulatory sequence of $X$, and $h_{X,T}$ is a non-linearity parameter representing cooperativity between multiple factors of the same type.

We use this equation to build a system of ordinary differential equations that describes the time evolution of concentrations of the relevant regulators $X$. Assume that the expression rate of $X$ is proportional to the activity of the regulatory site (defined by Eq. 1) as the average fraction of time the site is occupied by an activator). Assume also that each gene product undergoes exponential decay, that is, its concentration decreases at a rate proportional to itself. Then the change in gene product concentration over time can be described as

2 $$\frac{d\left[ X \right]}{{dt}} = \alpha_{X} SA\left( X \right) - \delta_{X} \left[ X \right],$$

where $\alpha_{X}$ and $\delta_{X}$ are scaling coefficients for the rate of transcription and decay, respectively (decay half-life is $\ln \left( 2 \right)/\delta_{X} )$.

This approach requires that Eq. (2), for each node $X$ in the network, depends only on other variables that are explicitly modelled in the GRN. In practice, most genes are influenced by other genes that are not present in the network because they are not considered relevant in the context of the GRN model. The influence of these interactions on the master equation of the quantitative model (Eq. 2) can be greatly simplified with a change of variables.

Considering Eq. (1), each constitutive inhibitor and activator $T$ will be represented by a term $\left( {[T] /k_{T,X} } \right)^{{h_{T,X} }}$ in the rate of expression $\alpha_{X} SA\left( X \right)$. Assuming that the concentration $\left[ T \right]$ is constant in the relevant conditions for the model, this term can be simplified to the constant value $\beta_{T,X} = \left( {[T] /k_{T,X} } \right)^{{h_{T,X} }}$. Furthermore, the combined influence of all constitutive activators is also constant $\beta_{act,X} = \sum\nolimits_{T \in act} {\beta_{T,X} }$, and for the constitutive inhibitors we have that their sum is equal to the constant $\beta_{inh,X} = \sum\nolimits_{T \in inh} {\beta_{T,X} }$. This leads to the equation

$$SA\left( X \right) = \frac{{\beta_{act,X} + \mathop \sum \limits_{{T \in act^{\prime}}} \left( {\frac{\left[ T \right]}{{k_{T,X} }}} \right)^{{h_{T,X} }} }}{{1 + \beta_{act,X} + \beta_{inh,X} + \mathop \sum \limits_{{T \in act^{\prime}}} \left( {\frac{\left[ T \right]}{{k_{T,X} }}} \right)^{{h_{T,X} }} + \mathop \sum \limits_{{T \in inh^{\prime}}} \left( {\frac{\left[ T \right]}{{k_{T,X} }}} \right)^{{h_{T,X} }} }},$$

where $act^{\prime}$ and $inh^{\prime}$ are the sets of non-constitutive activators and inhibitors. By dividing the numerator and denominator by $1 + \beta_{inh,X}$ and defining $\beta_{X} = \beta_{act,X} /\left( {1 + \beta_{inh,X} } \right)$, and $k_{T,X}^{^{\prime}} = k_{T,X} \left( {1 + \beta_{inh,X} } \right)^{{h_{T,X} }}$, the parameter set of the equation can be further reduced:

3 $$SA\left( X \right) = \frac{{\beta_{X} + \mathop \sum \limits_{{T \in act^{\prime}}} \left( {\frac{\left[ T \right]}{{k_{T,X}^{^{\prime}} }}} \right)^{{h_{T,X} }} }}{{1 + \beta_{X} + \mathop \sum \limits_{{T \in act^{\prime}}} \left( {\frac{\left[ T \right]}{{k_{T,X}^{^{\prime}} }}} \right)^{{h_{T,X} }} + \mathop \sum \limits_{{T \in inh^{\prime}}} \left( {\frac{\left[ T \right]}{{k_{T,X}^{^{\prime}} }}} \right)^{{h_{T,X} }} }}.$$

For nodes which are not activated by non-constitutive factors, the background parameter $\beta_{X}$ is also redundant. By dividing the numerator and denominator by $1 + \beta_{X}$ and defining $\alpha_{X}^{^{\prime}} = \alpha_{X} \beta_{X} /\left( {1 + \beta_{X} } \right)$ and $k_{T,X}^{^{\prime\prime}} = k_{T,X}^{^{\prime}} \left( {1 + \beta_{X} } \right)^{{h_{T,X} }}$ we obtain the expression

4 $$\alpha_{X}^{^{\prime}} \frac{1}{{1 + \mathop \sum \limits_{{T \in inh^{\prime}}} \left( {\frac{\left[ T \right]}{{k_{T,X}^{^{\prime\prime}} }}} \right)^{{h_{T,X} }} }}.$$

Henceforth we will not write the prime $^{\prime}$ on these transformed variables.

The Shea–Ackers formalism is derived with the assumption that all activations and inhibitions are physically realised by binding competition on a gene's regulatory sequence. The rate equations above assume that there is transcription at a constant rate whenever an activator is bound, thus ignoring differences in the rate at which transcription factors recruit the transcription machinery. Overlooking the complexity of different regulatory mechanisms allows for a mathematical approximation that can describe a wide range of dynamic behaviours that are realistic for different gene interaction mechanisms while using only a small number of parameters.

#### Viral inhibition and activation

For REST and miRs, formula (3) or (4) must also take into account the externally added factors shREST and viral Ascl1. The direct effect of shREST on the system is inhibition of REST. According to the GRN we use as a basis for our models, the effect of viral Ascl1 is the activation of miRs. Therefore, we could model the influence of these factors as a constant inhibitor acting on REST ($\beta_{{{\text{RESTi}}}}$) and a constant activator acting on miRs ($\beta_{{{\text{vAscl1}}}}$). These values are parameters of the model and are added to the denominator of $SA\left( {{\text{REST}}} \right)$, or the numerator and denominator of $SA\left( {{\text{miRs}}} \right)$, respectively.

These parameters $\beta_{{{\text{RESTi}}}}$ and $\beta_{{{\text{vAscl1}}}}$ can be added or removed from the model depending on whether the external influence they represent is present. Thus, the same model equations can represent the three stages of the experiment: (a) the fibroblast stage (i.e. $\beta_{{{\text{RESTi}}}} = \beta_{{{\text{vAscl1}}}} = 0$), (b) the three days of REST inhibition ($\beta_{{{\text{RESTi}}}} \neq 0$) and (c) the conversion stage, where both parameters are non-zero. The value of the parameters when not fixed to zero is trained to the data in the same way as other model parameters.

Note that viral influences are modelled as constant with respect to time, because the measured values are at least $10^{5}$ times greater than the baseline value for fibroblasts during the time frame under consideration, and the system behaviour shown in Fig. 1c does not suggest a fluctuating regulatory influence of Ascl1.

#### Model equations

Following this procedure to construct one ordinary differential equation (ODE) for each factor in the network yields an ODE system that can be numerically integrated to yield a time evolution of concentration levels $f_{{p,c_{0} }}^{X} \left( t \right)$ for each factor $X$, given initial concentration values $c_{0} = f_{{p,c_{0} }}^{X} \left( 0 \right)$ and an appropriate parameter set $p = \left\{ {\alpha_{X} ,\delta_{X} ,\beta_{X} } \right\}_{X} \cup \left\{ {k_{T,X} ,h_{T,X} } \right\}_{T,X} \cup \left\{ {\beta_{RESTi},\beta_{vAscl1}} \right\}$. For the initial literature-based model, the full ODE system is given in Eq. (5).

5 $$\left\{ {\begin{array}{*{20}l} {\frac{d}{dt}\left| {\left[ {\text{P}} \right]} \right| = \alpha_{{\text{P}}} \frac{1}{{1 + \left( {\left[ {\text{M}} \right]/k_{{{\text{M}},{\text{P}}}} } \right)^{{h_{{{\text{M}},{\text{P}}}} }} }} - \delta_{{\text{P}}} \left[ {\text{P}} \right]} \hfill \\ {\frac{d}{dt}\left| {\left[ {\text{N}} \right]} \right| = \alpha_{{\text{N}}} \frac{1}{{1 + \left( {\left[ {\text{M}} \right]/k_{{{\text{M}},{\text{N}}}} } \right)^{{h_{{{\text{M}},{\text{N}}}} }} + \left( {\left[ {\text{P}} \right]/k_{{{\text{P}},{\text{N}}}} } \right)^{{h_{{{\text{P}},{\text{N}}}} }} }} - \delta_{{\text{N}}} \left[ {\text{N}} \right]} \hfill \\ {\frac{d}{dt}\left| {\left[ {\text{M}} \right]} \right| = \alpha_{{\text{M}}} \frac{{\beta_{{\text{M}}} + \beta_{{{\text{vAscl1}}}} }}{{1 + \beta_{M} + \beta_{{{\text{vAscl1}}}} + \left( {\left[ {\text{R}} \right]/k_{{{\text{R}},{\text{M}}}} } \right)^{{h_{{{\text{R}},{\text{M}}}} }} }} - \delta_{{\text{M}}} \left[ {\text{M}} \right]} \hfill \\ {\frac{d}{dt}\left| {\left[ {\text{R}} \right]} \right| = \alpha_{{\text{R}}} \frac{{\beta_{{\text{R}}} + \left( {\left[ {\text{P}} \right]/k_{{{\text{R}},{\text{N}}}} } \right)^{{h_{{{\text{R}},{\text{N}}}} }} }}{{1 + \beta_{{\text{R}}} + \left( {\left[ {\text{P}} \right]/k_{{{\text{P}},{\text{R}}}} } \right)^{{h_{{{\text{P}},{\text{R}}}} }} + \left( {\left[ {\text{M}} \right]/k_{{{\text{M}},{\text{R}}}} } \right)^{{h_{{{\text{M}},{\text{R}}}} }} + \beta_{{{\text{RESTi}}}} }} - \delta_{{\text{R}}} \left[ {\text{R}} \right]} \hfill \\ {\frac{d}{dt}\left| {\left[ {\text{A}} \right]} \right| = \alpha_{{\text{A}}} \frac{1}{{1 + \left( {\left[ {\text{R}} \right]/k_{{{\text{R}},{\text{A}}}} } \right)^{{h_{{{\text{R}},{\text{A}}}} }} }} - \delta_{{\text{A}}} \left[ {\text{A}} \right]} \hfill \\ \end{array} } \right.$$

where each node in the GRN is indicated by its initial letter for brevity. For the nPTB $\to$ PTB model, we have for the first (PTB) equation:

6 $$\frac{d}{dt}\left[ {\text{P}} \right] = \alpha_{{\text{P}}} \frac{{\beta_{P} + \left( {\left[ {\text{N}} \right]/k_{{{\text{N}},{\text{P}}}} } \right)^{{h_{{{\text{N}},{\text{P}}}} }} }}{{1 + \beta_{P} + \left( {\left[ {\text{M}} \right]/k_{{{\text{M}},{\text{P}}}} } \right)^{{h_{{{\text{M}},{\text{P}}}} }} + \left( {\left[ {\text{N}} \right]/k_{{{\text{N}},{\text{P}}}} } \right)^{{h_{{{\text{N}},{\text{P}}}} }} }} - \delta_{{\text{P}}} \left[ {\text{P}} \right]$$

and an identical formula for the other equations, though with different parameter values after training. Trained parameter values are shown in Supplementary Table 2 for the literature model and nPTB $\to$ PTB model. These and all other models presented in this work are given in SBML format in the online supplementary.

As detailed above, these equations can be modified by removing the terms $\beta_{{{\text{RESTi}}}}$ and $\beta_{{{\text{vAscl1}}}}$ as necessary to represent the presence or absence of these external factors. Initial values for our simulations are given in Supplementary Table 3.

#### Genetic algorithm for parameter fitting

An ODE system is considered a good match for the data if there is some parameter set $p$ that leads to predicted expression curves $f_{{p,c_{0} }}$ that closely match observations. Finding such a parameter set is an optimization problem. We obtained the best results using a real-valued genetic algorithm, evolving a vector of real numbers which each represent a system parameter. The algorithm used a population size of 100, uniform crossover, and a mutation operator that modifies a small number of genes by multiplying with log-normally distributed random variables. Specifically, if $m(p,\sigma$) represents a random distribution that has a probability $\left( {1 - p} \right)$ of being $1$ and a probability $p$ of being distributed as $\exp \left( {{\mathcal{N}}\left( {0,\sigma } \right)} \right)$, then mutation was implemented by multiplying each variable with independent random values from the distribution $m\left( {2/n, 1.01} \right) \times m\left( {2/3n, 1.05} \right) \times m\left( {1/5n,1.5} \right)$, where $n$ is the number of genes. The initial population was composed of individuals with genes chosen uniformly from the unit interval, but more complex models were also run using parameters from simpler models as initial values.

Each real-valued gene represents a system parameter. Hill parameters $h_{T,X}$ were constrained to a maximum value of $4$ by transforming the gene value $h_{T,X}^{^{\prime}}$ to $h_{T,X} = 4\left( {h_{T,X}^{^{\prime}} } \right)/\left( {1 + h_{T,X}^{^{\prime}} } \right)$, where $h_{T,X}$ is the parameter value used in the system equations. The other parameters were used directly as they are in the real-valued vector genome. This means they are unbounded (though the mutation operator will never produce non-positive values).

#### Objective function for parameter fitting

The optimisation procedure is designed to yield ‘good’ parameter sets, according to a pre-defined measure. To quantify this, we used the parameter set to integrate the model equations as specified above in “Model equations”, and compare the outcome to data using a standard sum of squared errors (SSE) with smoothness regularisation. Specifically, the model equations were integrated three times, representing the fibroblast (denote the resulting curve with $f_{{p,{\text{fibro}}}}$), REST inhibition ($f_{{p,{\text{RESTi}}}}$), and conversion ($f_{{p,{\text{conv}}}}$) stages of the experiment. Each curve has its own set of initial values and slightly modified parameter set (see Viral inhibition and activation and Supplementary Table 3).

The objective function, which measures the quality of a parameter set $p$, consisted of several terms. The SSE term measures the difference between the system solution $f_{{p,{\text{conv}}}}$ and our observed data points during the first five days of conversion termed X in the following equation. The data values used here are the median values of the triplicate observations in each of the six time points. For the fibroblast stage, no error is included in this term; for the REST inhibition stage, the final value after three days is compared to the observed value; and for the conversion stage, all data points in the first five days are compared to the simulated values.

7 $$SSE = \mathop \sum \limits_{X} \left( {\left( {f_{{p,{\text{RESTi}}}}^{X} \left( {3{\text{ days}}} \right) - \left[ X \right]_{0} } \right)^{2} + \mathop \sum \limits_{{t \in \left\{ {8{\text{ hours}},1,2,3,5{\text{ days}}} \right\}}} \left( {f_{{p,{\text{conv}}}}^{X} \left( t \right) - \left[ X \right]_{t} } \right)^{2} } \right).$$

A pure SSE approach often resulted in highly oscillating fits, which closely matched the data but fluctuated erratically in between. To obtain more realistic solutions, we added a smoothness regularisation term of the form:

8 $$R_{1} = \lambda \mathop \sum \limits_{X} \mathop \smallint \limits_{{\text{5 days}}} \left| {\frac{{d^{2} }}{{dt^{2} }}f_{{p,{\text{conv}}}}^{X} \left( t \right)} \right|dt.$$

Finally, an important property of the system being modelled is that fibroblasts do not spontaneously convert to neurons, i.e. the mRNA concentrations should remain stable when initialized near the observed fibroblast baseline and when not perturbed by the external terms $\beta_{{{\text{RESTi}}}}$ and $\beta_{{{\text{vAscl1}}}}$. This is enforced with another term in the fitness function, punishing any changes in concentration during a three-day simulation of the ${\text{fibro}}$ stage.

9 $$R_{2} = \mu \mathop \sum \limits_{X} \mathop \smallint \limits_{{\text{3 days}}} \left| {\frac{d}{dt}f_{{p,{\text{fibro}}}}^{X} \left( t \right)} \right|dt.$$

We obtained best results with $\lambda = \frac{1}{20}$ and $\mu = \frac{1}{20}$. In the evolutionary algorithm, parameter sets with a higher fitness are considered better, so the fitness is set equal to the negative value $- \left( {SSE + R_{1} + R_{2} } \right)$.

Two nodes in our networks represent a combination of more than one gene for which expression was measured. We fitted the miRs network node to transcription data from miR-124, and the RESTc network node to data from the REST gene. This arbitrary choice did not influence the results due to the high correlation between the concentration values (this was verified by using mRNA concentration levels of miR-9/9* and SCP1 with identical results).

### Deterministic simulation

Integration of the ODE system was done using a custom implementation of the fourth-order Runge–Kutta algorithm during training, with a time step size of 0.05 h. Trained models were integrated and plotted using Mathematica. Solutions were verified to be identical using both methods.

### Stochastic simulation

Stochastic simulations were performed using a custom implementation of the Gillespie algorithm^25^. Noise levels were adjusted by modifying the reaction formulas: the stoichiometric coefficients were multiplied by a noise level $\eta$, and reaction rates correspondingly divided by $\eta$ to maintain the same expected number of molecules added or removed per time unit. Our data is a bulk average and therefore does not suggest a realistic value for $\eta$. In the figures shown here (Figs. 4b,c, 6a), its value was set to $0.1$. However, two adjustments were made. First, PTB and REST have a (relative) concentration range close to $1$, whereas the other factors have typical concentration levels closer to $5$. For this reason, transcription and decay reactions for these two factors were transformed with an $\eta$ that was five times lower. Second, these same factors also tend to fit with much higher reaction rates, i.e. both higher $\alpha$ and $\delta$ in (2). To obtain more similar noise levels, $\eta$ for these reactions was adjusted by an additional factor of $1/5$, resulting in a total of $\eta = 0.004$ for PTB and REST, and $\eta = 0.1$ for nPTB, miRs and Ascl1.

Overexpression and knockdown were simulated by modifying the appropriate transcription rate coefficient. Specifically, for the overexpression, resp. knockdown, of a factor $X$, the corresponding transcription rate coefficient $\alpha_{X}$ was multiplied by $5$, resp. $1/5$. Overexpression of Ascl1 and knockdown of miRs were instead controlled by multiplying the values $\beta_{{{\text{RESTi}}}}$ and $\beta_{{{\text{vAscl1}}}}$ with 0 or 1, in the same way as when training the models. In the case of PTB knockdown, lack of conversion was also verified when $\alpha_{{{\text{PTB}}}}$ was multiplied by × $1/5000$, and at this rate conversion was observed when the background activation of REST was multiplied by $1/5$.

### Overexpression and knockdown simulations

A simulation was considered a neuronal conversion when endogenous expression of Ascl1 was reached at least 1 (in units corresponding to the detection limit of the qPCR experiment) at some point during the simulation. This was taken to indicate that the system switched to a different state that could be self-sustaining when the externally induced viral expression of Ascl1 would be removed. An OX/KD scenario was considered to cause neuronal conversion when at least one of fifty performed simulations showed these criteria. These thresholds are arbitrary, but the result that the nPTB $\to$ PTB model is the best model to predict experimental outcomes is robust.

### Measuring relative influence of interactions

In order to break down the total transcription rate of each factor into contributions from its activators and inhibitors, the terms in the numerator and denominator of the transcription rate Eq. (1) were calculated at each time point of a trajectory and plotted, as in Figs. 3d and 6b. Activators and inhibitors are represented by arrows pointing upwards and downwards, respectively, with arrow length showing the relative magnitude of that interaction term.

Specifically, the total length of all activation arrows $L_{X}^{act}$ of a gene $X$, at some time point $t$, equals the fraction of the maximum transcription rate that would be realized if no inhibitors were present. The total length of the inhibition arrows $L_{X}^{inh}$ is defined so that the difference between the activation and the inhibition arrows equals the realized transcription activity $SA\left( X \right)$ (Eq. 1). This results in the following equations:

10 $$\left\{ {\begin{array}{*{20}l} {L_{X}^{act} { = }\frac{{{\Sigma }_{act} }}{{1 + {\Sigma }_{act} }}} \hfill \\ {L_{X}^{inh} = \frac{{{\Sigma }_{act} {\Sigma }_{inh} }}{{\left( {1 + {\Sigma }_{act} } \right)\left( {1 + {\Sigma }_{act} + {\Sigma }_{inh} } \right)}},} \hfill \\ \end{array} } \right.$$

where $\Sigma_{act} = \sum\nolimits_{T \in act} {\left( {\left[ T \right]/k_{T,X} } \right)^{{h_{T,X} }} }$ and $\Sigma_{inh} = \sum\nolimits_{T \in inh} {\left( {\left[ T \right]/k_{T,X} } \right)^{{h_{T,X} }} }$ (see Eq. 1). Given these total lengths for activator and inhibitor arrows, individual activators and inhibitors $T$ are given lengths proportional to their current magnitude $\left( {\left[ T \right]/k_{T,X} } \right)^{{h_{T,X} }}$, so that the contribution of each inhibitor or activator to the total transcription rate becomes visible.

Finally, an arrow is added to represent the decay term in the Eq. (2), with a length of $\frac{{\delta_{X} }}{{\alpha_{X} }}\left[ X \right]$ (essentially, the decay rate in the same units of $\alpha_{X}$ as the other arrows). By adding this additional arrow, the difference between the sum of upward and downward pointing arrows shows the rate of change in mRNA concentrations (Eq. 2 divided by $\alpha_{X}$).

## Supplementary Information

Supplementary Information.

## Data Availability

The SBML implementation of the models is available at: https://github.com/victorahnell/Direct-Neural-Conversion-Models-SBML/blob/main/code.zip.
