# Supplementary material for: A quantitative model of cellular decision making in direct neuronal reprogramming
Source: Sci Rep. 2021 Jan 15;11:1514. doi: 10.1038/s41598-021-81089-8 (PMC7810861; doi:10.1038/s41598-021-81089-8)
Supplement: Supplementary file 1 — Supplementary Information. [file 41598_2021_81089_MOESM1_ESM.pdf]

Supplementary Information

A Quantitative Model of Cellular Decision Making in Direct Neuronal Reprogramming

Adriaan Merlevede[1], Emilie M. Legault[3], Viktor Drugge[1], Roger A. Barker[2], Janelle Drouin-Ouellet[3,*], Victor Olariu[1,*]

[1]Computational Biology and Biological Physics, Department of Astronomy and Theoretical Physics, Lund University, Lund, 223 63, Sweden

[2] Cambridge Centre for Brain Repair, University of Cambridge, Forvie Site, Robinson Way, Cambridge CB2 2PY, UK.

[3]Faculté de Pharmacie, Université de Montréal, Montréal, Québec, H3T 1J4, Canada

[*]Correspondence: victor.olariu@thep.lu.se, janelle.drouin-ouellet@umontreal.ca

qRT-PCR analysis

See main Methods section for details.

Supplementary Table 1: List of primers used for qRT-PCR analysis

Gene	Direction	Sequence (5' → 3')
ACTB	Forward	CCTTGCACATGCCGGAG
	Reverse	GCACAGAGCCTCGCCTT
GAPDH	Forward	TTGAGGTCAATGAAGGGGTC
	Reverse	GAAGGTGAAGGTCGGAGTCA
YWHAZ	Forward	GAAGCATTGGGGATCAAGAA
	Reverse	AGCAGATGGCTCGAGAATACA
REST	Forward	AAATGTGGCCTTAACTGGGGAA
	Reverse	TCTGTCTTTCTTCACCGACCAG
PTB	Forward	GGGTCGGTTCCTGCTATTCC
	Reverse	AAAAGCTCGTCAGATCCCCG
nPTB	Forward	TGGTTACGCCCCAAAGTCTG
	Reverse	TGGTTTCCATCAGCCATCTGT
Endogenous Ascl1	Forward	CTAAAGATGCAGGTTGTGCG
	Reverse	GGAGCTTCTCGACTTCACCA
Viral Ascl1	Forward	CATCTCCCCCAACTACTCCA
	Reverse	TCTGGGCTAAGAGGGTCGTA

	Literature	nPTB → PTB
$resti$	1.25	3.17
$viral$	0.793	0.813
α_P	0.0889	18.8
$k_{M,P}$	0.000387	0.0306
$h_{M,P}$	0.154	1.63
α_N	179.	18.1
$k_{M,N}$	7.88e6	1.44e4
$h_{M,N}$	2.65	3.99
$k_{P,N}$	0.181	0.203
$h_{P,N}$	4.	4.
α_M	0.384	6.05
$k_{R,M}$	2.14e-17	0.529
$h_{R,M}$	0.0000195	2.63
β_M	0.0555	0.402
α_R	0.0934	9.68
$k_{P,R}$	3.81e7	0.561
$h_{P,R}$	0.0000825	4.
$k_{M,R}$	4.4	3.5
$h_{M,R}$	4.	4.
β_R	3.53	9.54
α_A	612.	5.28
$k_{R,A}$	0.0711	0.123
$h_{R,A}$	4.	4.
δ_P	0.0185	0.138
δ_N	0.0797	0.0177
δ_M	0.0102	0.374
δ_R	0.0765	9.32

	Literature	nPTB → PTB
δ_A	0.245	0.0042
$k_{N,P}$	-	1.63
$h_{N,P}$	-	4.
β_P	-	2.02

Supplementary Table 2: Parameter values for the literature and PTB → nPTB model, see equations 5 and 6. Parameters and equations for other models are provided in online supplement.

Supplementary Table 3: Initial values of simulations in the three stages of the experiment. The values are normalized so that a value of 1 is equal to the fibroblast level, or to the detection limit in the case of Ascl1, which was not observed in fibroblasts.

Experiment stage	PTB	nPTB	REST	miRs	Ascl1
Fibroblast	1	1	1	1	0
REST inhibition	1	1	1	1	0
Conversion	0.831538	1.27675	1.08673	0.645663	0

Supplementary Figures

A Quantitative Model of Cellular Decision Making in Direct Neuronal Reprogramming

Adriaan Merlevede[1], Emilie M. Legault[3], Viktor Drugge[1], Roger A. Barker[2], Janelle Drouin-Ouellet[3,*], Victor Olariu[1,*]

[1]Computational Biology and Biological Physics, Department of Astronomy and Theoretical Physics, Lund University, Lund, 223 63, Sweden

[2] Cambridge Centre for Brain Repair, University of Cambridge, Forvie Site, Robinson Way, Cambridge CB2 2PY, UK.

[3]Faculté de Pharmacie, Université de Montréal, Montréal, Québec, H3T 1J4, Canada

[*]Correspondence: victor.olariu@thep.lu.se, janelle.drouin-ouellet@umontreal.ca

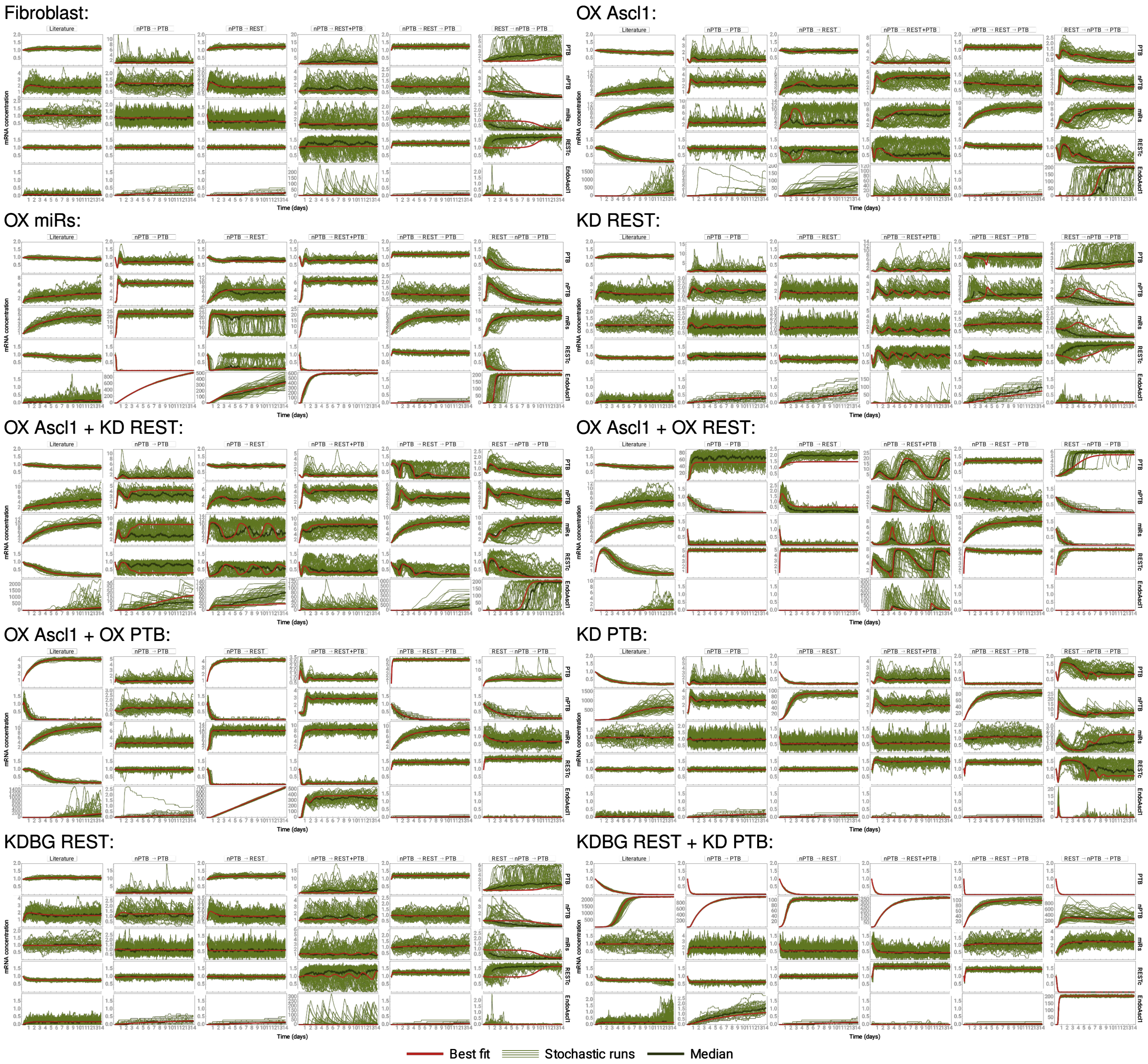

Supplementary Figure 1: ***Simulated overexpression and knockdown experiments in several performant models;*** *accompanying Figure 4.*

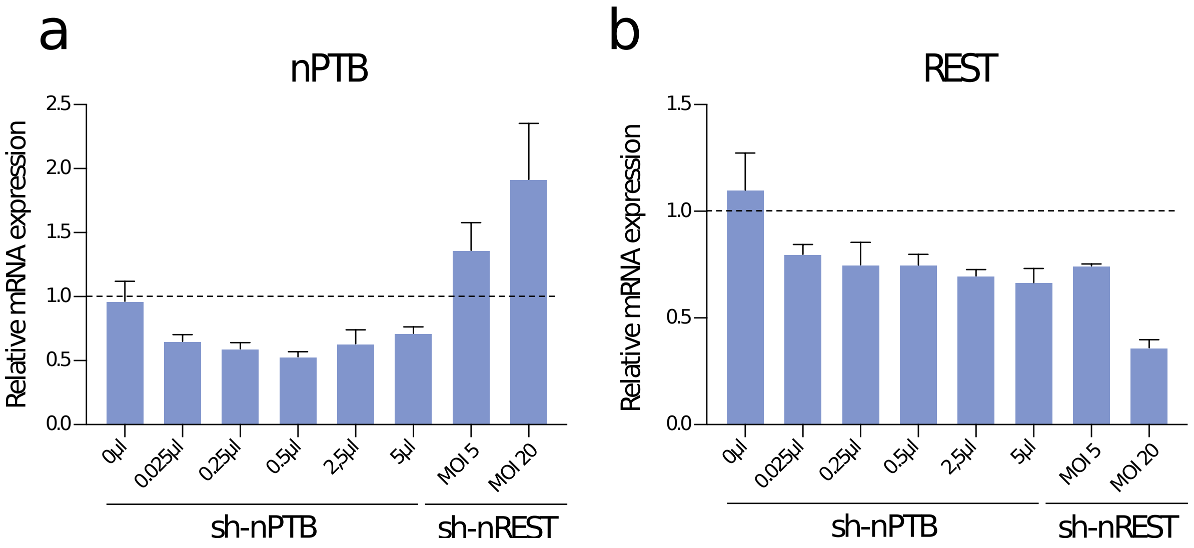

Supplementary Figure 2: **nPTB and REST expression following KD with shRNAs**. (**a**) Measured transcription levels of nPTB 3 days following infection with different volumes or MOI of lentiviruses containing either shRNAs against nPTB or REST. The measurements are normalized so that a value of 1 is equal to the fibroblast level (represented as a dashed line). Error bars are defined as S.D. (**b**) Measured transcription levels of REST 3 days following infection with different volumes or MOI of lentiviruses containing either shRNAs against nPTB or REST. The measurements are normalized so that a value of 1 is equal to the fibroblast level (represented as a dashed line). Error bars are defined as S.D.
